# Hydrological model parameter regionalization: Runoff estimation using machine learning techniques in the Tha Chin River Basin, Thailand

**DOI:** 10.1016/j.mex.2024.102792

**Published:** 2024-06-07

**Authors:** Phyo Thandar Hlaing, Usa Wannasingha Humphries, Muhammad Waqas

**Affiliations:** a The Joint Graduate School of Energy and Environment (JGSEE), King Mongkut's University of Technology Thonburi (KMUTT), Bangkok, 10140, Thailand; b Center of Excellence on Energy Technology and Environment (CEE), Ministry of Higher Education, Science, Research and Innovation, Bangkok, Thailand; c Department of Mathematics, Faculty of Science, King Mongkut's University of Technology Thonburi (KMUTT), Bangkok, 10140, Thailand

**Keywords:** Hydrological modeling, SWAT, Regionalization, Machine Learning, Ungauged basin, Advancements in Daily Precipitation Prediction

## Abstract

Understanding hydrological processes necessitates the use of modeling techniques due to the intricate interactions among environmental factors. Estimating model parameters remains a significant challenge in runoff modeling for ungauged catchments. This research evaluates the Soil and Water Assessment Tool's capacity to simulate hydrological behaviors in the Tha Chin River Basin with an emphasis on runoff predictions from the regionalization of hydrological parameters of the gauged basin, Mae Khlong River Basin. Historical data of Mae Khlong River Basin from 1993 to 2017 were utilized for calibration, followed by validation using data from 2018 to 2022.

• Calibration results showed the SWAT model's reasonable accuracy, with R² = 0.85, and the validation with R² of 0.64, indicating a satisfactory match between observed and simulated runoff.
• Utilizing Machine Learning (ML) techniques for parameter regionalization revealed nuanced differences in model performance. The Random Forest (RF) model exhibited an R² of 0.60 and the Artificial Neural Networks (ANN) model slightly improved upon RF, showing an R² of 0.61 while the Support Vector Machine (SVM) model demonstrated the highest overall performance, with an R² of 0.63.
• This study highlights the effectiveness of the SWAT and ML techniques in predicting runoff for ungauged catchments, emphasizing their potential to enhance hydrological modeling accuracy. Future research should focus on integrating these methodologies in various basins and improving data collection for better model performance.

## Graphical abstract

**Figure:**
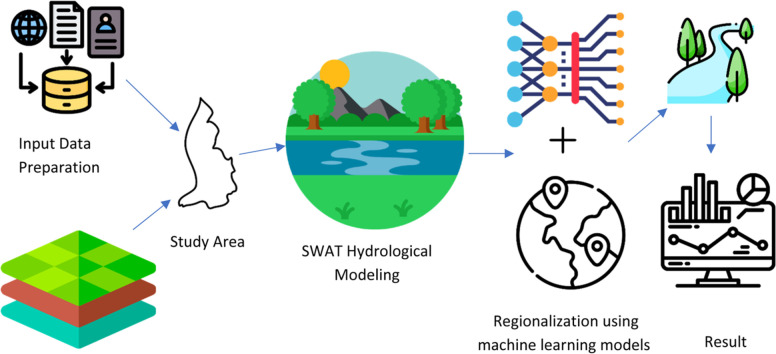

Specifications table

**Table:** 

Subject area:	Engineering
More specific subject area:	*Modeling and Forecasting*
Name of your method:	*Advancements in Daily Precipitation Prediction*
Name and reference of original method:	*NA.*
Resource availability:	*Data used to support the study's findings can be obtained from the corresponding author upon request.*

## Background

Hydrological models serve as valuable instruments for simulating hydrological processes, finding widespread utility in flood prediction, water resource administration, and evaluating the repercussions of climate variations in current times [1,2]. Streamflow is a critical component of hydrological modeling [3], and accurate streamflow assessment for effective water resource management is essential, especially in areas where flooding or water scarcity are challenges [4]. Accurately predicting streamflow requires advanced modeling approaches due to the inherent complexity of hydrological systems, further exacerbated by anthropogenic effects and climate unpredictability [5]. Over recent decades, hydrological models have emerged as the predominant method for predicting runoff [6,7]. Nevertheless, these models impose calibration of free parameters using observed discharge data before forecasting runoff hydrographs, data requirements unmet in numerous catchment areas [8]. Prediction of streamflow in ungauged catchments is challenging for hydrologists [9]. Traditional hydrological models, like the Soil and Water Assessment Tool (SWAT), have been instrumental in providing insights into water balance and predicting streamflow [10]. In 2020, Xue et al. investigated various regionalization strategies to study several hydrological models for runoff prediction in gauged and ungauged basins. [11]. Pawan Upadhyay (2022) highlighted the utility of the SWAT model in the field of hydrology for simulating complex water systems, highlighting its significance for water resource decision-making [12]. Over the past decade, numerous hydrologists have explored different methodologies to estimate data for ungauged basins. Guo, Zhang et al. (2021) conducted a review of different regionalization techniques for hydrological models, emphasizing the advantages, disadvantages, and difficulties of using these methods in catchments that are not gauged [1]. To predict daily streamflow at ungauged basins in Turkey, Yilmaz and Onoz (2020) used three statistical methods: the inverse similarity weighted (ISW) approach, the multiple-donor stations drainage area ratio (MDAR) technique, and the single donor station drainage area ratio (DAR) method. [13]. A review by Belvederesi in 2022 focused on river flow focusing, modeling issues unique to certain regions, and locations with insufficient hydrological observations [14]. Regionalization refers to the technique of forecasting runoff in ungauged basins by transferring knowledge from gauged to ungauged catchments [15]. Regionalization, by incorporating regional watershed information, such as land cover, soil moisture, and hydrological groups from gauged to ungauged basins [16]. Regionalization strategies seek to overcome the inherent difficulties of modeling in the lack of direct observational data by transferring hydrological knowledge from data-rich (gauged) to data-poor (ungauged) locations. Merz (2004) looked at the processes for converting the parameters of hydrological models from gauged catchments to ungauged catchments and the author found that the multiple regression method is better than the spatial proximity approach for catchment attributes [17]. Oudin (2008) assessed different regionalization approaches for hydrological modeling in ungauged catchments. They compared methods through the use of day for spatial proximity (using data from neighboring catchments), physical similarity (identifying catchments with comparable features), and regression (developing relationships between catchment attributes and streamflow) [18]. While, Wu (2022) explored how catchment characteristics (e.g., size, topography, land cover) can inform regionalization, the broader integration of regionalization methods with SWAT and ML techniques remains underexplored [19]. Existing methodologies, Senent-Aparicio (2023) highlighted, yield mixed results due to the complex interplay of hydrological processes across different scales [20]. The literature indicates a growing consensus on the need for novel regionalization approaches that can leverage the strengths of both process-based and data-driven models [21]. Ditthakit (2023) assessed the usage of ML and the GR2M model for monthly runoff prediction in Southern Thailand [22]. However, studies that systematically compare the effectiveness of these approaches in a cohesive framework, particularly within the Thailand hydrological context, are still scarce. Parallel to hydrological models, ML techniques have emerged as powerful tools for hydrological prediction, offering the potential to enhance or even outperform traditional models in accuracy and efficiency [23]. Models such as Artificial Neural Networks (ANN), Random Forest (RF), and Support Vector Machines (SVM) were identified by Salmasi (2021). The non-linear relationships between hydrological inputs and outputs underscore their potential in streamflow prediction [24]. Thailand, with its diverse climatic zones and complex river systems, presents a unique case for the study of streamflow estimation [25,26]. The Mae Khlong and Tha Chin River Basins are critical watersheds in Thailand, serving diverse ecological functions, supporting agriculture, and providing water resources to urban and rural communities. Accurate hydrological modeling is crucial for sustainable water management, predicting floods, and mitigating drought impacts in these basins [27]. Despite the advancements in hydrological and ML techniques, several gaps remain in the literature, particularly regarding the comparative analysis of these models across different hydrological and climatic contexts. Both the advantages and disadvantages of ML and SWAT models have been shown in studies. A crucial gap, nonetheless, remains in the lack of direct comparisons across these models in various geographical contexts, particularly in Southeast Asia [28,29]. Furthermore, the application of these models in ungauged basins, such as the Tha Chin River basin, is underexplored, presenting significant challenges in model parameterization and validation due to the lack of direct streamflow observations. The Tha Chin River basin is an ungauged basin in central Thailand, which is particularly significant due to its agricultural productivity and susceptibility to both floods and droughts in Thailand. The existence of the Tha Chin River basin in low land area and the effect of tidy water, made the study area lack the observation of river data. Due to the data scarcity problem and the topography problem, most of the previous studies in the Tha Chin River basin have been done only for some areas of the study. A study by Yasin in 2014 used the SWAT hydrological model to investigate flow, water quality, and sediment issues in the upper Tha Chin River basin [30]. Suphanburi Province is in the middle section of the Tha Chin River basin. Monprapussorn (2018) investigated the impacts of climate change and land-use changes on the basin's water resources [31]. Existing reviews of previous research in the Tha Chin River basin have identified a notable gap in comprehensive streamflow studies covering the entire basin and forecasting streamflow. This study aims to address this gap by providing a comprehensive framework for investigating streamflow estimation through hydrological modeling, integrating advanced ML-based regionalization techniques.

This study aims to investigate streamflow estimation through the integration of hydrological modeling and watershed regionalization. The unique contribution of this research lies in its comprehensive examination of the performance of both SWAT and ML techniques, combined with advanced regionalization techniques, across gauged (Mae Khlong) and ungauged (Tha Chin) basins. The study's outcomes offer valuable insights into the applicability of hydrological models and hold promise for informing the development of resilient and adaptive water resource management strategies in regions grappling with data scarcity and hydrological variability. By systematically comparing and integrating various modeling approaches, this research underscores the significance of innovative regionalization strategies in enhancing the precision and dependability of streamflow predictions.

## Method details

**Fig. 1:**
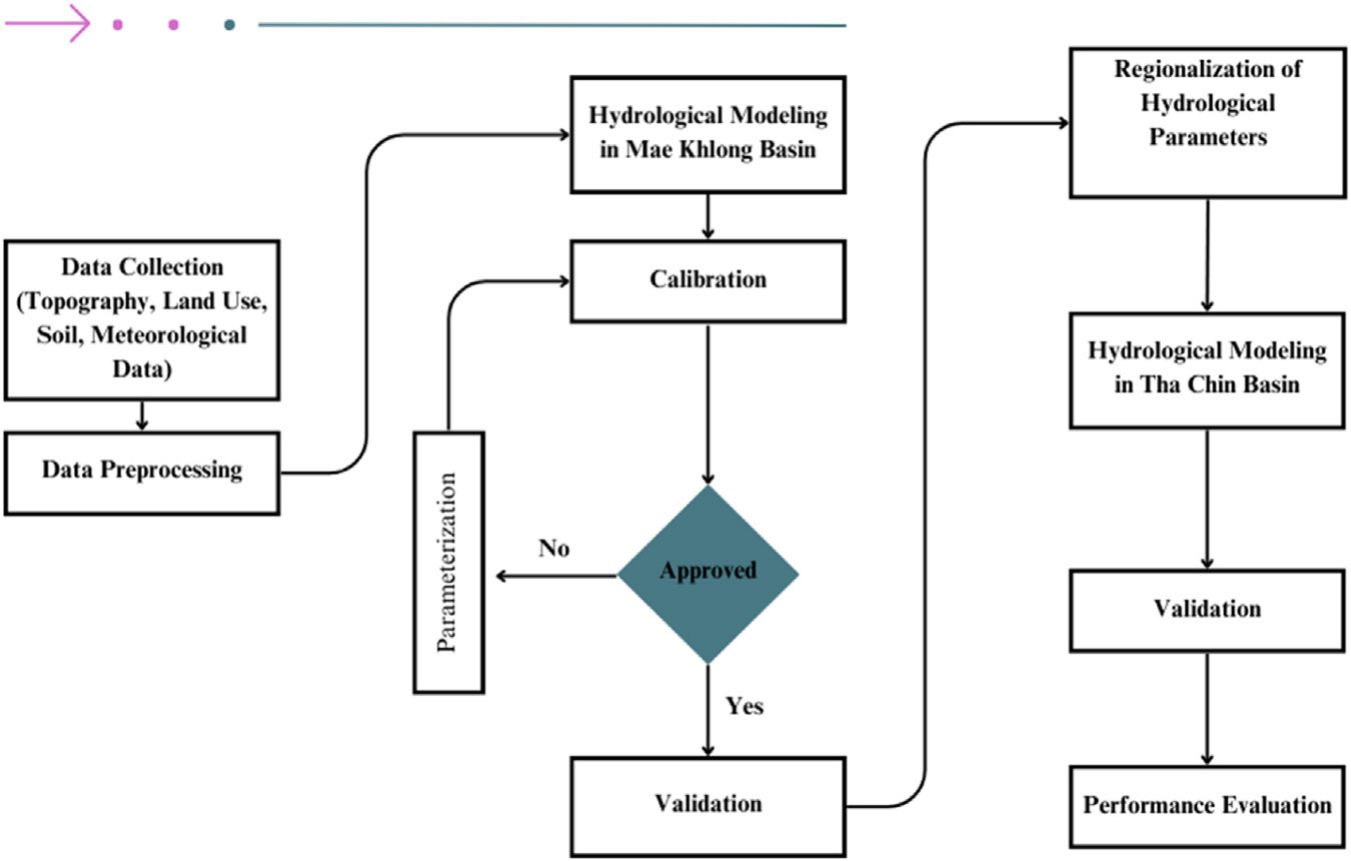
Overall methodology employed in this study.

The study used a three-step approach to achieve its primary goal, as shown in Fig. 1, which serves as the research's methodological flowchart.

### Study area

**Fig. 2:**
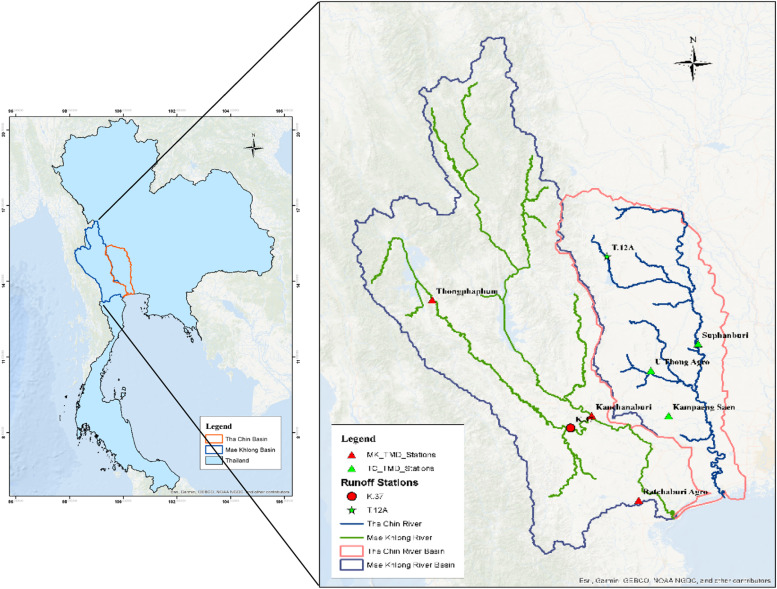
The location map of Mae Khlong River Basin and Tha Chin River Basin.

**Fig. 3:**
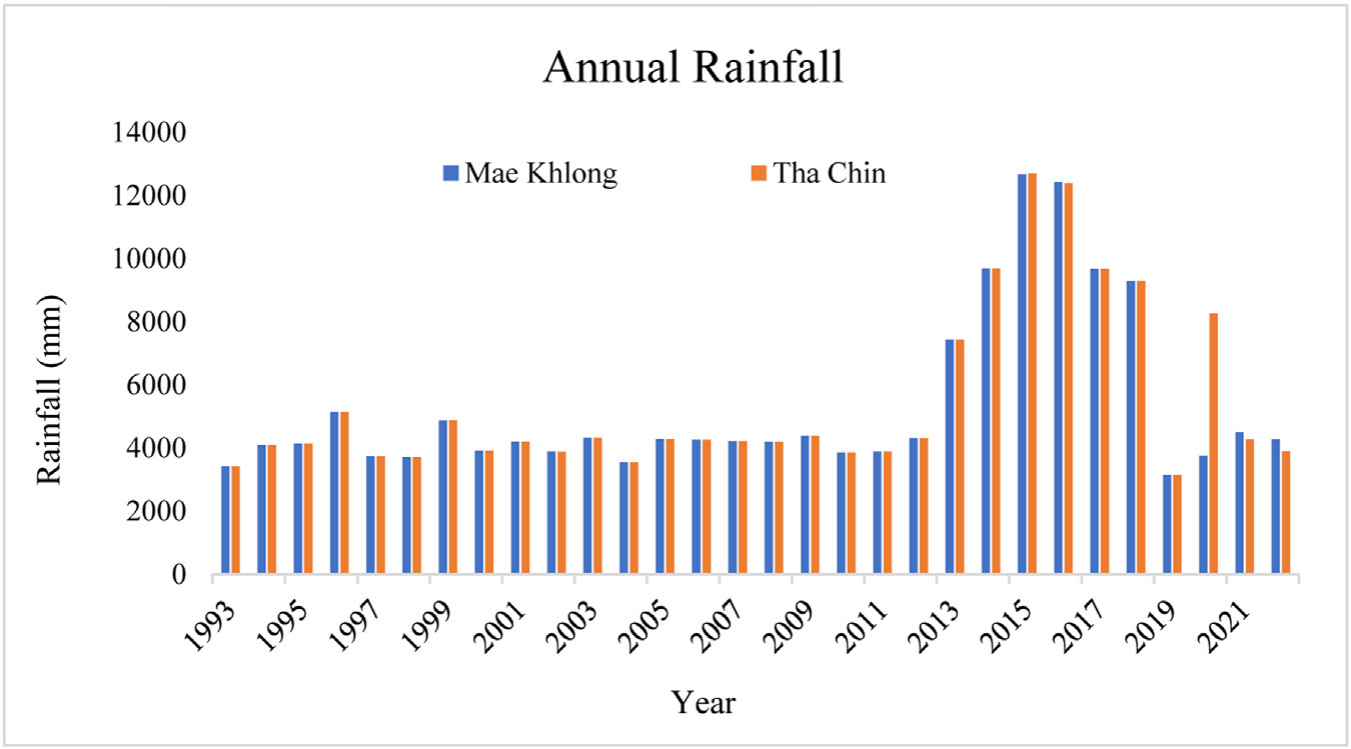
Rainfall pattern in Mae Khlong Basin and Tha Chin Basin.

The study encompasses two critical river basins in Thailand: the Mae Khlong serving as a gauged basin and the Tha Chin River basin, identified as an ungauged basin. The Mae Khlong basin is located in Western Thailand, covering an area of approximately 30,000 square kilometers, with elevations ranging from sea level to over 1500 m in its mountainous regions [32]. The Tha Chin River basin, a distributary of the Chao Phraya River, lies in the central plain of Thailand, covering an area of nearly 14,000 square kilometers, with an elevation from sea level to over 1300 m [33]. Fig. 2 represents the location of the Mae Khlong River Basin and the Tha Chin River Basin and their hydrological and meteorological stations. Thailand's climate is primarily characterized as tropical monsoon, featuring a wet season (May to October) and a dry season (November to April). The selected basins are in the central part of Thailand having similar climatic conditions with an average annual rainfall of 1200–1600 mm, with temperature fluctuations of 19 °C to 38 °C. Particularly, seasonal variations exert a significant influence on river flow volumes, with peak flows typically observed during the monsoon season [34]. Fig. 3 shows the annual rainfall for the Mae Khlong Basin and the Tha Chin River basin.

### Data collection

**Fig. 4:**
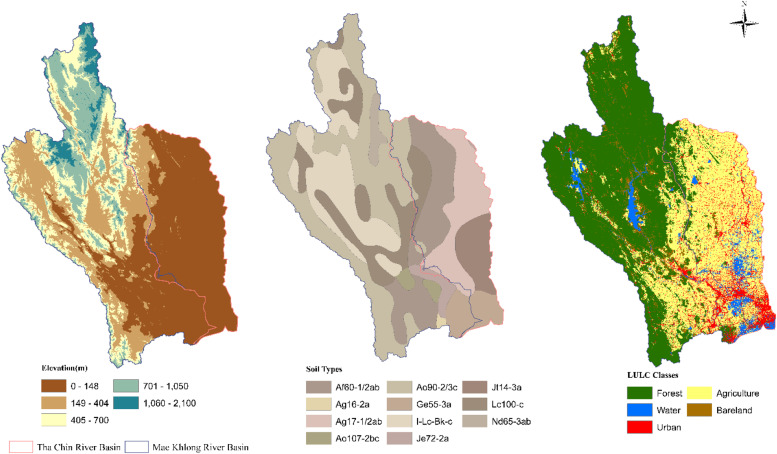
(a) Digital elevation model map (b) Soil map (c) Land use land cover map for the Mae Khlong River Basin and the Tha Chin River Basin.

The hydrological datasets essential for modeling and analysis were meticulously compiled for both the gauged basin, the Mae Khlong River Basin, and the ungauged basin, the Tha Chin River Basin. These datasets were curated to encompass a comprehensive array of meteorological, hydrological, and physical attributes, ensuring the precision of streamflow simulation and prediction within these basins. Meteorological datasets, encompassing precipitation, temperature, wind speed, and relative humidity data, were collected for 1993 to 2022, these datasets boast a daily temporal resolution. Acquired from satellite weather stations, the data underwent rigorous validation against international standards by the Thai Meteorological Department (TMD). Daily streamflow records for the gauged basin were procured from the Royal Irrigation Department (RID). However, given the absence of direct streamflow observations in the Tha Chin River basin, it is classified as an ungauged basin for this study. To address this data gap, regionalization techniques were employed, facilitating the transfer of hydrological insights from gauged basins to estimate streamflow patterns within the Tha Chin basin. For the characterization of physical data attributes, a 30m-resolution Digital Elevation Model (DEM) for the study was acquired from Copernicus Global and the European Digital Elevation Model (COP-DEM) developed by the European Space Agency as shown in Fig. 4(a)**.** Land use data crucial for this study were extracted from the ESRI Global Land Cover Map, renowned for its detailed 10 m resolution, providing comprehensive insights into land use patterns across the basins shown in Fig. 4(b) and (c). Furthermore, soil property data were sourced from the Digital Soil Map of the World (DSMW), distinguished by its scale resolution of 1:50,000. These collected data were integrated into the SWAT modeling framework, allowing for the precise and accurate calculation of streamflow and other relevant hydrological variables. All data types and sources are given in Table 1.

**Table 1:** Data types and sources.

Data Types	Data Sources	Remark
Meteorological Data (Precipitation, Temperature, Relative Humidity, Wind Speed)	Thai Meteorological Department (TMD)	1993–2022
Digital Elevation Models (DEM) and Topographical data	Open Topography https://opentopography.org	30-m resolution Copernicus DEM [35]
Soil Data (texture, hydraulic properties, and so on)	Digital Soil Map of the World (DSMW) https://data.fao.org/DSMW	1:5.000.000
Land Use/Land Cover Data	Sentinel-2 10 m Land Use/Land Cover https://www.argis.com	2017–2022
Streamflow Data	Royal Irrigation Department (RID), Thailand	1993–2022

### SWAT hydrological modeling

SWAT is built on a foundation of multiple physical equations that simulate the hydrologic cycle, sediment transport, crop growth, nutrient cycling, and land management practices [36]. One of the core components of SWAT is the water balance equation, which is central to the simulation of hydrological processes:

(1) $$SW_{t}=\;SW_{0}+\;\sum_{i=1}^{t}\left(R_{day}-Q_{surf}-ET_{a}-W_{seep}-Q_{gw}\right)$$

Where *SW_t_* is the final soil water content(mm), SW_0_ is the initial water content on day i (mm), *R_day_* precipitation on day i(mm), *Q_surf_* surface runoff on day i(mm), *ET_a_* evapotranspiration on day i(mm), *W_seep_* water entering the vadose zone from the soil profile on day i(mm), and *Q_gw_* return flow on day i(mm) [37].

**Fig. 5:**
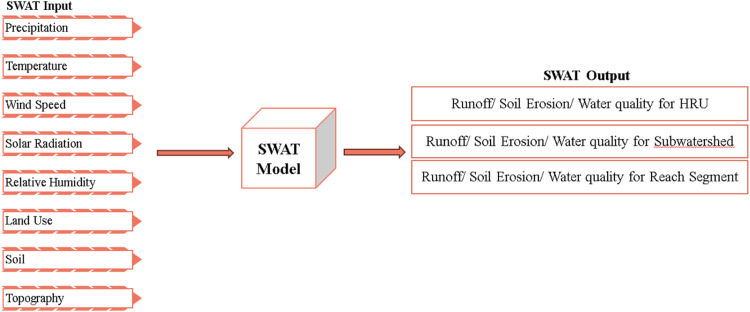
Overview of SWAT model – model input/output [37].

The model was set up using the ArcSWAT interface for ArcGIS. The basin was delineated into 27 sub-basins based on the digital elevation model (DEM), and the hydrological response units (HRUs) were defined by overlaying land use, soil, and slope data. The model's calibration and uncertainty analysis were conducted using the SUFI-2 algorithm within the SWAT Calibration and Uncertainty Programs (SWAT-CUP). Sequential Uncertainty Fitting (SUFI-2) was chosen for its robustness in quantifying both parameter uncertainty and the uncertainty in model outputs. The calibration process involved adjusting key model parameters to match the simulated streamflow with observed data over a specific period 1993–2022. The overall methodology for the SWAT model is shown in Fig. 5.

### Model performance evaluation

The performance of the calibrated model was evaluated in SWAT-CUP using the Nash-Sutcliffe Efficiency (NSE), the Coefficient of Determination (R²), Kling Gupta Coefficient (KGE), the Ratio of the Root Mean Square Error to the Standard Deviation of measured data (RSR). and the Percent Bias (PBIAS) during both calibration and validation periods [38]. The detailed equations are as follows:

(2) $$NSE=1-\;\frac{\sum_{i=1}^{n}{\left(Q_{sim}-Q_{obs}\right)}^{2}}{\sum_{i=1}^{n}{\left(Q_{obs}-Q_{avg,obs}\right)}^{2}}$$

(3) $$R^{2}={\left[\frac{\sum_{i=1}^{n}\left(Q_{obs}-Q_{avg,obs}\right)\left(Q_{sim}-Q_{\mathrm{avg},sim}\right)}{\sqrt{\sum_{i=1}^{n}{\left(Q_{obs}-Q_{avg,obs}\right)}^{2}}\;.\sqrt{\sum_{i=1}^{n}{\left(Q_{sim}-Q_{avg,sim}\right)}^{2}}}\right]}^{2}$$

(4) $$PBIAS=\left(\frac{\sum_{i=1}^{n}\left(Q_{obs}-Q_{sim}\right)}{\sum_{i=1}^{n}Q_{obs}}\right)\;x\;100$$

(5) $$RSR=\;\frac{\sqrt{\sum_{i=1}^{n}{\left(Q_{obs}-Q_{sim}\right)}^{2}}}{\sqrt{\sum_{i=1}^{n}{\left(Q_{obs}-Q_{avg,obs}\right)}^{2}}}$$

(6) $$KGE=1-\;\sqrt{{\left(r-1\right)}^{2}+{\left(\alpha -1\right)}^{2}+\;{\left(\beta -1\right)}^{2}}$$

$$r=\;\frac{\sigma_{sim}/\mu_{sim}}{\sigma_{obs}/\mu_{obs}}\;\alpha =\;\frac{\sigma_{sim}}{\sigma_{obs}}\;\beta =\;\frac{\mu_{sim}}{\mu_{obs}}$$

Where, Q_obs_, represents the observed discharge, and Q_sim_, is the simulated discharge. Additionally, metrics such as Q_avg,obs_ and Q_avg,sim_ refer to the average observed and simulated discharge, respectively. Furthermore, σ_sim_ and σ_obs_ represent the standard deviation of simulated and observed discharge, respectively, providing insights into the variability of discharge data. Finally, μ_sim_ and μ_obs_ signify the mean values of simulated and observed discharge, respectively [38].

### Regionalization implementation

Regionalization in hydrology assists the extrapolation of hydrological data from gauged to ungauged basins, using physical, climatic, and hydrological properties as proxies to build prediction correlations in areas lacking direct observations [40,41]. Regionalization methods include parametric transfer, spatial closeness, physical resemblance, and statistical or ML techniques [42]. Parametric transfer adjusts model parameters based on catchment features, whereas spatial proximity uses data from surrounding basins with similar hydrological responses. Physical similarity detects catchments with similar properties, and statistical or ML techniques detect patterns in datasets, training models on gauged basin data to predict variables in ungauged basins [17,43].

**Fig. 6:**
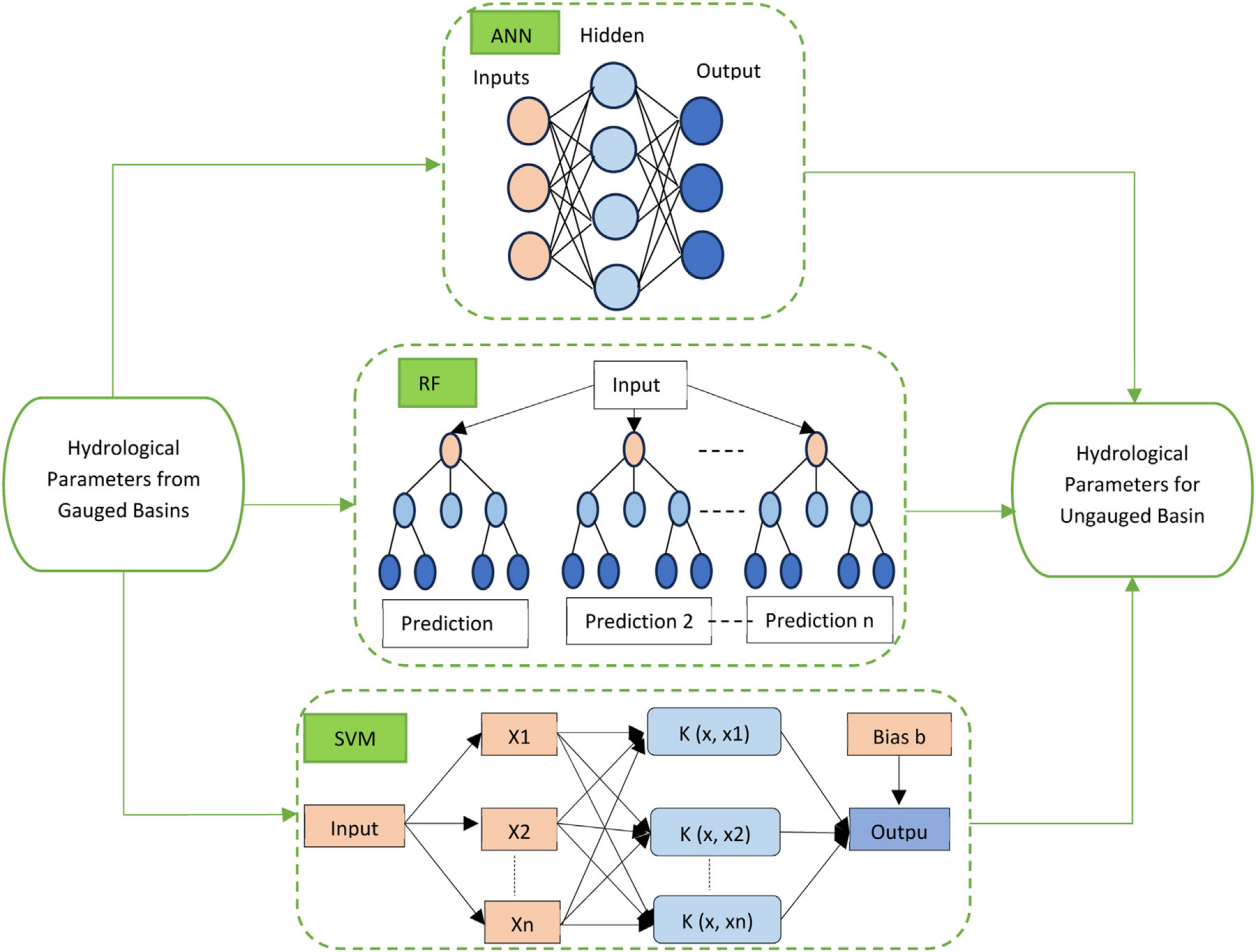
Application of machine learning models for parameter prediction.

In this study, the regionalization process is operationalized using ML techniques, which use catchment features as predictors of streamflow in ungauged basins (Fig. 6). Unlike traditional methods that rely on direct hydrological measurements, spatial closeness, or physical resemblance, ML techniques can deal with complicated, non-linear correlations and interactions between numerous catchment features and hydrological responses. Supervised learning models are trained on datasets from gauged basins that contain both predictors (catchment attributes) and response variables (for example, streamflow, and flood frequency). The selection of supervised learning models was guided by the specific characteristics of our research question and dataset. Commonly used models include ANNs, SVMs, and RF. Models are evaluated using techniques such as cross-validation to guarantee their predicted performance generalizes effectively to unknown data [23].

The selection of ML techniques (ANN, SVM, RF) for hydrological parameter regionalization is strategically driven by the research objectives, data characteristics, and available computational resources. If high predictive accuracy is paramount, ANNs are favored, while RFs are preferred when understanding feature importance is crucial. Model choice also depends on whether the task is classification (SVM) or regression (ANN, RF), the size and dimensionality of the dataset, and its quality. While ANNs have the potential for very good performance, they can be prone to overfitting. SVMs and RFs, on the other hand, consistently offer good performance and are less susceptible to overfitting [44]. Model performance evaluations are determined by using different evaluation metrics such as R2, NSE, and so on, with the performance ranges shown in Table 2.

**Table 2:** Performance ratings for the evaluation metrics [39].

Rating	NSE	R^2^	KGE	PBIAS	RSR
Very Good	0.75<NSE<1.00	0.7 < R^2^<1.0	0.75<KGE <1	|PBIAS|< 10 %	RSR<=0.5
Good	0.65<NSE<0.75	0.5 < R^2^<0.7	0.65<KGE<0.75	10 %<|PBIAS|< 15 %	0.5<RSR<0.6
Satisfactory	0.5<NSE<0.65	0.5 < R^2^<0.4	0.5<KGE<=0.65	15 %<|PBIAS|<25 %	0.6<RSR<0.7
Unsatisfactory	NSE < 0.5	R^2^ < 0.4	KGE <0.5	|PBIAS| > 25 %	RSR > 0.7

The study utilized calibrated and validated SWAT model parameters within each ML framework. The spatial interpolation method, inverse distance weighting, was employed for parameter regionalization, estimating values across ungauged areas using data from gauged basins. This process is crucial for extrapolating parameter values accurately. Additionally, a cross-validation technique was applied to ensure the reliability and generalization capability of regionalized parameters. This involved partitioning the dataset into subsets, iteratively training the model on one subset and validating it on another, and assessing performance and generalization to unseen data, thus enhancing the accuracy of regionalization.

### Sensitivity analysis

This study employs SWAT-CUP for sensitivity analysis to identify key parameters influencing hydrological simulations like streamflow, sediment yield, or nutrient loading. SWAT-CUP integrates tools for sensitivity analysis, calibration, validation, and uncertainty analysis. Global Sensitivity Analysis (GSA) within SWAT-CUP assesses parameter influences across their entire range via a multiple regression system, utilizing Latin hypercube-generated parameters.

(7) $$g=\;\alpha +\sum_{i=1}^{m}\beta_{i}.b_{i}$$

A *t*-test is then used to identify the relative significance of each parameter bi. In the analysis, the larger in absolute value, the value of t-stat, and the smaller the p-value, the more sensitive the parameter is. In SUFI-2, sensitivity analysis is part of the iterative process of calibration and uncertainty quantification. Parameters are ranked based on their influence on the output variance, guiding the calibration toward the most significant parameters [38].

## Method validation

### SWAT modeling for Mae Khlong River Basin

**Fig. 7:**
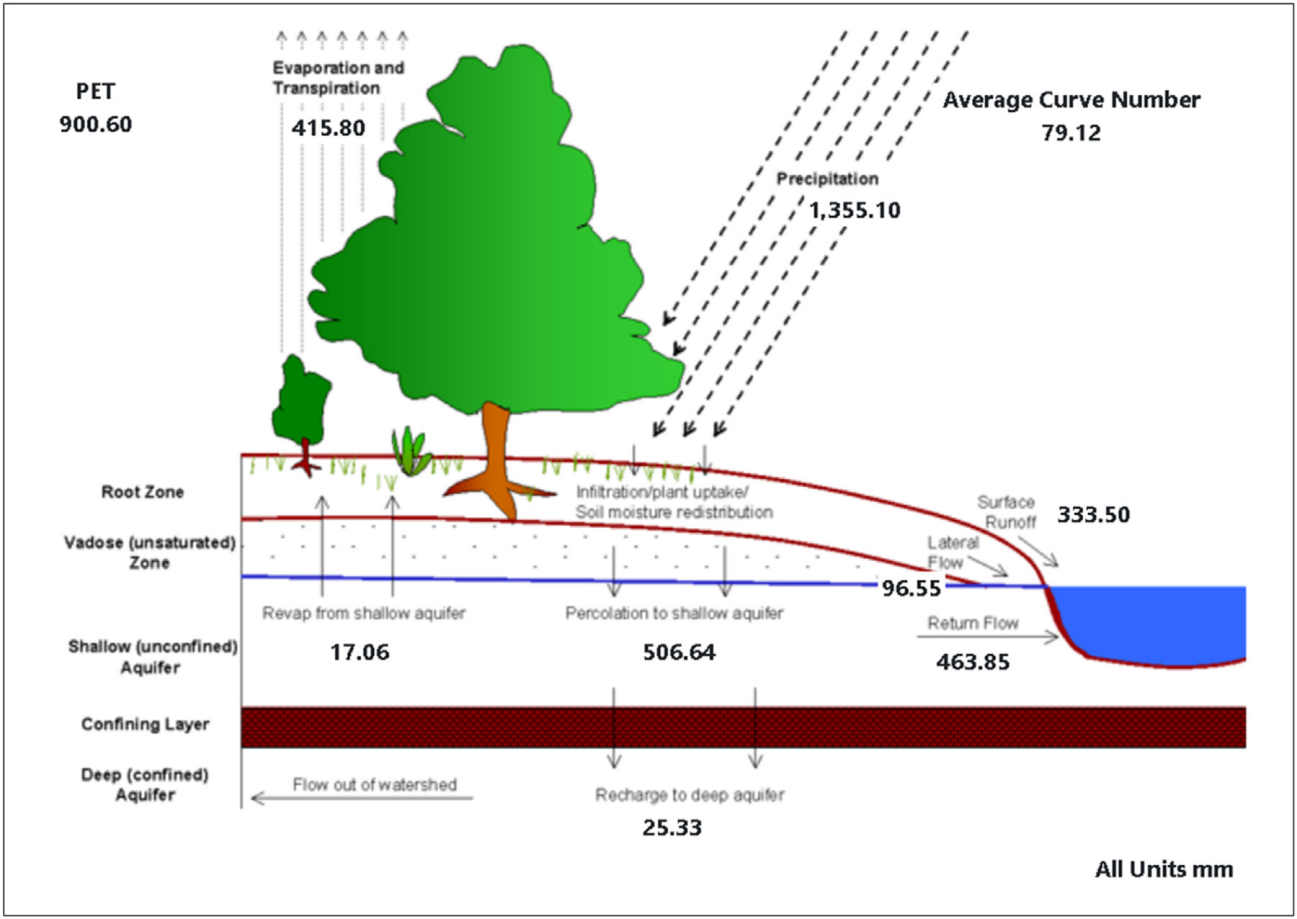
SWAT model output.

This study used SWAT to simulate hydrological processes in the Mae Khlong River Basin, with a focus on runoff analysis. To begin, the river basin was divided into sub-watersheds, which were then classified into Hydrologic Response Units (HRUs) based on land use, soil types, and slope differences for complete investigation as shown in Fig. 7.

**Fig. 8:**
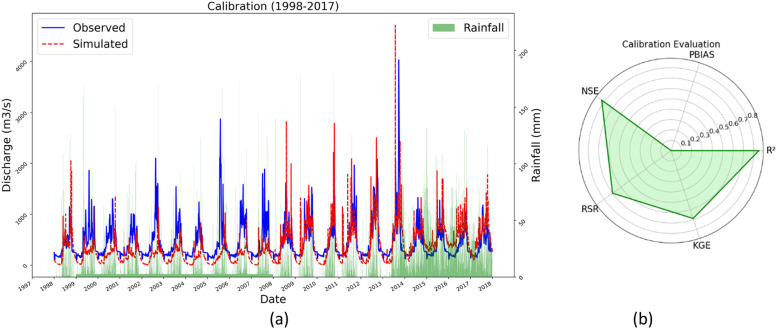
(a)Calibration of observed and simulated discharge in Mae Khlong River Basin. (b) Calibration evaluation.

**Fig. 9:**
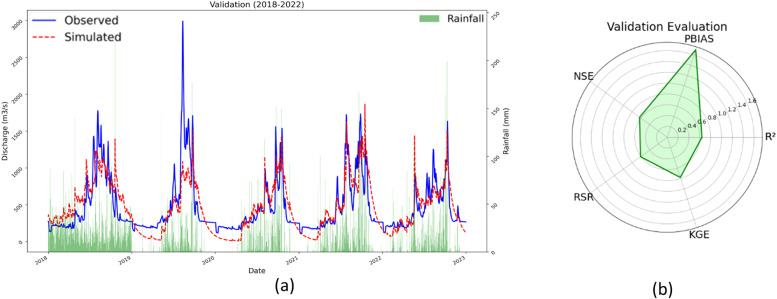
(a) Validation of observed and simulated discharge in Mae Khlong River Basin. (b) Validation evaluation.

The calibration process involved modifying specific hydrological parameters within SWAT, such as curve number, baseflow parameters, and soil parameters, to match observed runoff data from several gauging stations in the Mae Khlong River Basin. Using SWAT-CUP, the Sequential Uncertainty Fitting (SUFI-2) technique allowed for iterative parameter adjustments, enhancing the model's predictive accuracy and reliability. Calibration used historical data from 1993 to 2017 with five-year warm-up intervals, whereas validation used data from 2018 to 2022 to ensure a thorough evaluation of the model's performance throughout time. The model was thoroughly evaluated during both the calibration and validation stages, with a focus on its capacity to reliably recreate observed runoff patterns in the Mae Khlong River Basin. Table 3 shows the calibration and validation results. During calibration, as shown in Fig. 8(a) and (b) the SWAT model showed reasonable accuracy with R² = 0.65, NSE = 0.63, PBIAS = −8.4 (slight underestimation), KGE = 0.69, and RSR = 0.71. The validation phase yielded similar results, with R² and NSE values of 0.64 and 0.63, respectively, indicating a satisfactory fit between observed and predicted runoff (Fig. 9(a) and (b)). Notably, the PBIAS improved to 1.7 during validation, indicating a more balanced prediction without substantial underestimation or overestimation. Furthermore, the KGE grew to 0.78 and the RSR reduced to 0.61, indicating that the model performed better during validation. These findings highlight the SWAT model's efficacy in reproducing the hydrological behavior of the Mae Khlong River Basin, which accurately captures seasonal fluctuations typical of the region's monsoon climate. Table 1 contains detailed data, whereas Fig. 7, Fig. 8 visually display the calibration and validation outcomes.

**Table 3:** Evaluation of model for Mae Khlong River Basin.

Phase	R^2^	NSE	PBIAS	KGE	RSR
Calibration	0.85	0.83	0.0	0.69	0.7
Validation	0.64	0.63	1.7	0.78	0.61

#### Calibrated and validated hydrological parameters

Key factors were found and fine-tuned during the hydrological modeling of the Mae Khlong River Basin with the SWAT model to improve the model's precision and dependability. The curve number (CN), which controls runoff volume and peak flow rates by combining soil type, land use, and moisture conditions, proved critical in simulating the basin's hydrological response. Furthermore, precise calibration of the baseflow alpha factor (ALPHA_BF) was critical for accurately representing groundwater input to streamflow dynamics. Parameters such as soil accessible water capacity (AWC) and plant uptake compensation factor (EPCO) were adjusted to provide an accurate portrayal of water balance components within the basin, given their major impact on runoff generation and evapotranspiration processes. These factors were chosen because of their significant impact on the hydrological cycle, emphasizing their critical significance in establishing robust and realistic model simulations. Table 4 contains detailed explanations of the calibration and validation parameters.

**Table 4:** Calibrated and validated parameters.

Parameter	Description	Initial range	Final range
CN2	Curve Number for moisture condition II	−0.2 to 0.2.	27 to 34
Alpha_BF	Baseflow alpha factor (days)	0 to 1	0.7 to 0.8
GW_Delay	Groundwater delay (days)	0 to 100	30 to 40
GWQMN	Treshold depth of water in the shallow aquifer required for return flow to occur (mm)	0 to 500	80 to 90
GW_Revap	Groundwater "revap" coefficient	0 to 1	0.17 to 0.18
Revapmn	Threshold depth of water in the shallow aquifer for "revap" to occur (mm)	0 to 100	51 to 52.2
ESCO	Soil evaporation compensation factor	0 to 1	0.8 to 1.1
EPCO	Plant uptake compensation factor	0 to 1	0.5 to 0.65
CH_K2	Effective hydraulic conductivity in main channel alluvium	−0.01 to 5	−3 to 2
Surlag	Surface runoff lag time	0.05 to 24	40 to 45
OV_N	Average slope length	0.01 to 1	1 to 1.5
Slubbsn	Average slope length	0 to 80	80 to 95
CH_N2	Manning's "n" value for the main channel	−0.01 to 0.3	0.3 to 0.4
Sol_AWC	Available water capacity of the soil layer	0 to 1	−0.8 to −0.5
SOL_K	Saturated hydraulic conductivity	0 to 500	20 to 30
PLAPS	Precipitation lapse rate	0 to 1000	100 to 200
TLAPS	Temperature lapse rate	0 to 1000	500 to 800

#### Sensitivity analysis of Mae Khlong River Basin

**Fig. 10:**
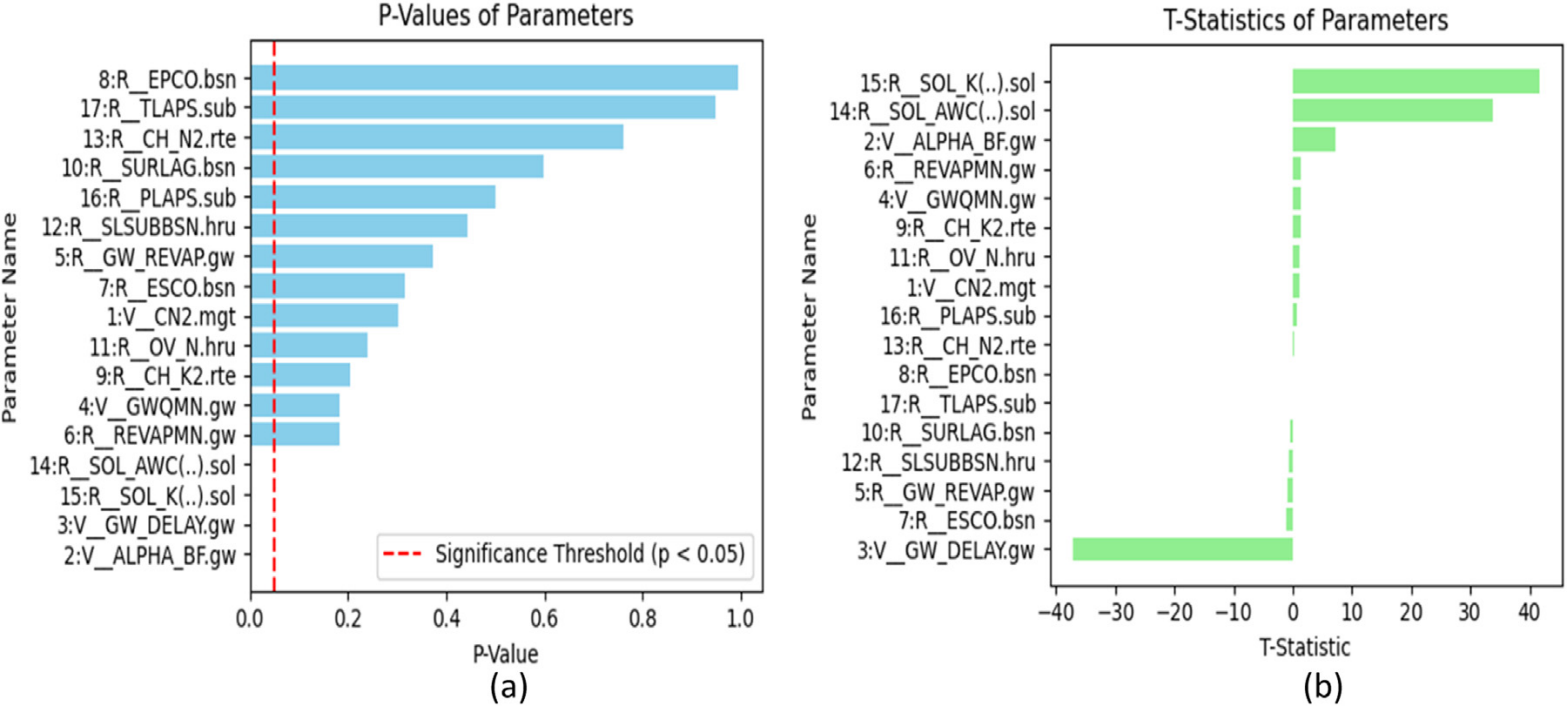
Sensitivity analysis of parameters for Mae Khlong River Basin (a)*P*-values (b) T-statistics.

The sensitivity analysis of the parameters from calibration and validation of the gauged Mae Khlong Basin was done using global sensitivity analysis in the SUFI2 algorithm of SWAT-CUP. The results in Fig. 10(b) show that the parameters, SOL_K and SOL-AWC are the most sensitive with 41.6 and 33.9 t-stat values and a p-value of 0, respectively, which means they have a critical influence on model performance. GW_Delay which has a negative t-stat of 37.1 delineates its critical influence on groundwater delay processes, highlighting the inverse relationship sharing with the model's performance metrics. Alternatively, EPCO and TLAPS, with t-stat values of 0.008 and −0.065, respectively, and p-values approaching 1, exhibit the least sensitivity on the model's output and can be negligible. Fig. 10(a) shows the p-values and t-stat analysis for the Mae Khlong River Basin to check the sensitivity of the SWAT model parameters.

### Hydrological modeling of Tha Chin river basin

In the cross-validation of the ungauged Tha Chin river basin, regionalized model parameters derived from three ML techniques —RF, ANN, and SVM were applied in SWAT hydrological modeling. The performance of these models was evaluated using key metrics including R², NSE, PBIAS, KGE, and RSR, as summarized in Table 5. The results indicate that all models achieved moderate to good predictive accuracy, with R² values ranging from 0.6 for RF to 0.63 for SVM, showing that the SVM model's superior predictive capability. NSE values also reflected satisfactory to good simulation accuracy, with SVM achieving the highest efficiency at 0.62. However, PBIAS values revealed a significant discrepancy in the ANN model, showing a substantial negative bias at −20, indicating a tendency to underestimate runoff values. In contrast, RF and SVM demonstrated acceptable bias levels within the expected range, with values of 5.49 and −7.5, respectively. The KGE metric further affirmed the superior performance of the ANN model with a value of 0.64, suggesting a better overall representation of the hydrological behavior compared to RF and SVM, which also performed well within or above the expected range. However, the RSR metric indicated that while RF and ANN remained within the acceptable range, suggesting reasonable error levels relative to observed data variability, the SVM model exceeded the upper limit with an RSR of 0.78, implying higher residual error. The SVM model exhibited the best performance in terms of predictive accuracy R², NSE, and KGE, despite having higher residual error RSR) and PBIAS compared to RF. The ANN model, while achieving high KGE, was significantly biased, which may affect the reliability of its predictions. The RF model offered a balanced performance with acceptable bias, efficiency, and error levels. These findings demonstrate the potential of ML techniques in enhancing hydrological modeling for ungauged basins, particularly highlighting the overall promising performance of the SVM model despite its higher error levels.

**Table 5:** Performance metrics of RF, ANN, SVM.

Metric	Expected range	RF	ANN	SVM
R^2^	0.4 – 1	0.6	0.61	0.63
NSE	0.5 - 1	0.55	0.6	0.62
PBIAS	−15 - 15	5.49	−20	−7.5
KGE	0.5 – 0.7	0.5	0.64	0.61
RSR	0.5 – 0.7	0.67	0.65	0.78

#### Regionalization by random forest

**Fig. 11:**
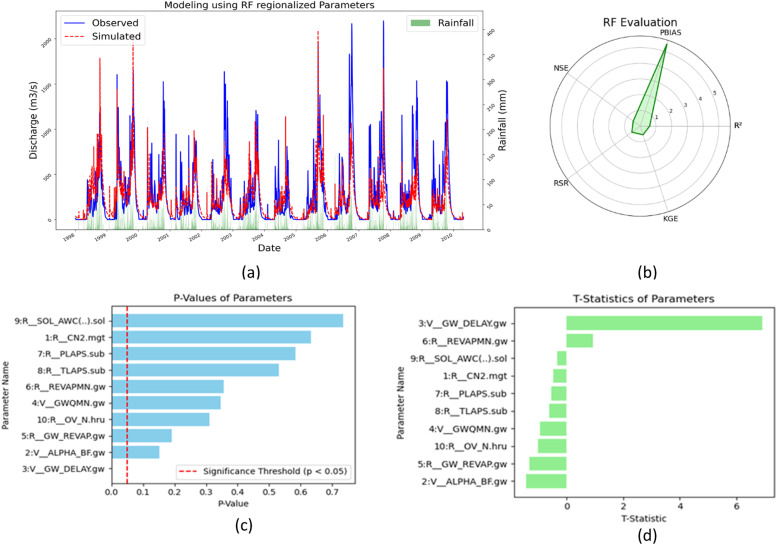
(a)Streamflow comparison (b) Model evaluation (c) *P*-values (d) t-stat.

Utilizing calibrated and validated SWAT model parameters within an RF regionalization framework, ten out of seventeen parameters were identified as suitable for regionalization (Fig. 11(c) and (d)). Evaluation of the model's effectiveness was conducted using key metrics including R², NSE, KGE, and RSR, resulting in satisfactory outcomes with values of 0.6, 0.55, 0.5, and 0.67, respectively (Fig. 11(b)). Subsequent sensitivity analysis of the regionalized parameters, employing t-statistics and p-values, discerned their influence on model performance. Parameters linked to groundwater and base flow, notably GW_DELAY, GWQMN, and Alpha_BF, were highly sensitive, evidenced by t-stat values approximating zero and *p*-values below 0.5. In contrast, parameters such as SOL_AWC, CN2, and evapotranspiration (PLAPS and TLAPS) exhibited lower sensitivity, with t-stat values ranging between −0.3 and −0.6 and p-values exceeding 0.5. The RF model's regionalization process highlighted GW_DELAY as the paramount parameter for accurate hydrological forecasting in the SWAT model application to the Tha Chin River basin. This analysis underscores the importance of meticulous parameter selection and sensitivity assessment in refining the accuracy of hydrological models within similar regional contexts.

#### Regionalization by ANN

**Fig. 12:**
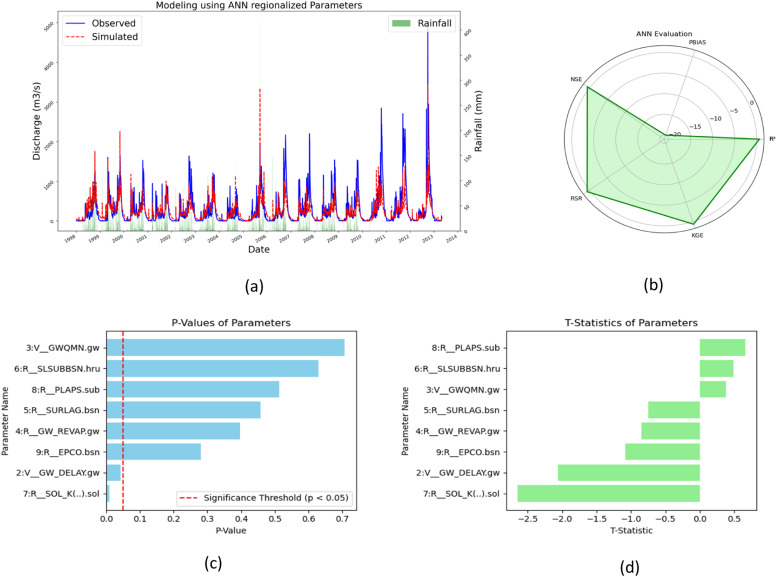
(a)Streamflow comparison (b) Model evaluation (c) *P*-values (d) t-stat.

Following validation, 17 parameters go through regionalization via an ANN approach, resulting in the identification of eight applicable parameters for basin modeling. The model's accuracy in predicting discharge was rigorously evaluated using hydrological performance indicators: R² = 0.63, NSE = 0.6, PBIAS = −20, KGE = 0.64, and RSR = 0.65, indicating satisfactory performance despite a tendency for underestimation reflected by the negative PBIAS value. Further sensitivity analysis using SUFI-2 revealed varying degrees of parameter sensitivity, with parameters like GWQMN, SLSUBBSN, and PLAPS showing lower sensitivity (t-stat values: 0.38 to 0.66, *p*-values: 0.51 to 0.71), while GW_DELAY and SOL_K exhibited higher sensitivity (t-stat values: −2.06 and −2.65, *p*-values: 0.04 and 0.01), signifying significant impacts on model performance. These findings are depicted in Fig. 12(a) to (d).

#### Regionalization by SVM

**Fig. 13:**
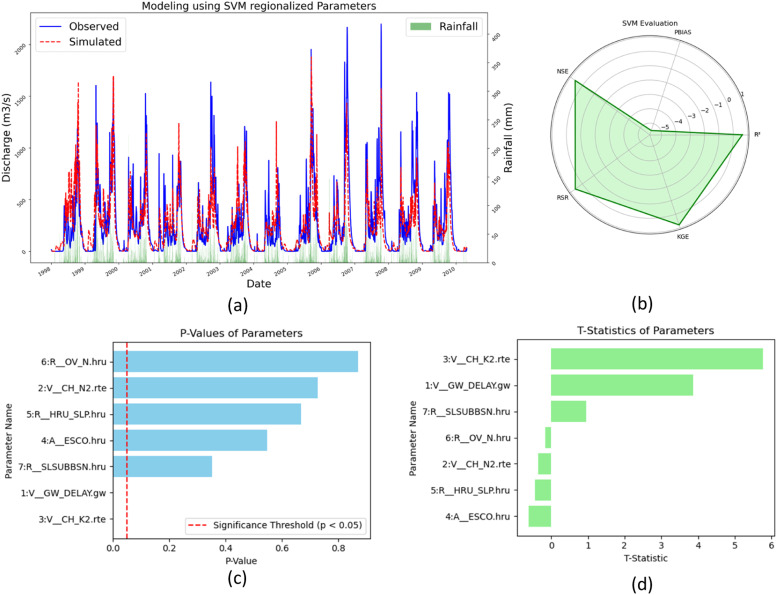
(a)Streamflow comparison (b) Model evaluation (c) *P*-values (d) t-stat.

The SWAT modeling performance utilizing parameters from SVM-based regionalization showcased an R² of 0.59, NSE of 0.59, and PBIAS of 7.5, indicating a satisfactory level of accuracy in flow prediction with a minor tendency towards underestimation. KGE and the RSR were recorded at 0.69 and 0.64, respectively, supporting the model's reliability. The standard deviations of 264.69 for simulated and 328.18 for observed flows suggest robust model performance, indicating potential for improvement in capturing the full variability of observed data. Sensitivity analysis of regionalized parameters using the SVM model highlighted parameters like GW_DELAY and CH_K2, demonstrating high sensitivity with t-stat values of 3.87 and 5.76, and p-values of 0.001 and 0.0001, respectively. Such high sensitivity underscores the critical role these parameters play in accurately simulating hydrological processes. Conversely, parameters like OV_N, CH_N2, HRU_SLP, and ESCO exhibited lower sensitivity, as indicated by their t-stat and p-value metrics. Evaluation results and sensitivity analyses are depicted in Fig. 13(a)–(d).

### Discussion

This study operationalizes the regionalization process using advanced ML techniques, which offer significant improvements over traditional methods by handling complex, non-linear relationships between catchment characteristics and hydrological responses. This approach aligns with recent advancements in the field, such as [30] stated the potential of ML models like ANNs, SVMs, and RF in enhancing predictive accuracy for ungauged basins. Our selection of these ML techniques was strategically driven by the specific characteristics of our dataset and research objectives, like the methodology employed by Arnold (2008) in their comprehensive study of hydrological modeling using SWAT [36]. findings reinforce and extend the claims made in these previous studies by demonstrating the effective use of ML-based regionalization to enhance the SWAT model's accuracy in the Tha Chin River Basin. Specifically, the SVM model showed superior predictive capability with an R² of 0.63, corroborating the evidence on the robustness of SVMs in hydrological predictions [31].

The incorporation of SWAT into the hydrological research of the Mae Khlong River Basin represents a significant step forward in understanding and controlling the complex hydrological processes in this region. This study methodically divides the river basin into sub-watersheds and HRUs, recognizing the variability in land use, soil types, and slope changes. Such rigorous segmentation is critical for accurately simulating hydrological responses and is consistent with approaches used in similar watershed studies (e.g., Qi et al., 2023), where dividing watersheds into HRUs significantly improved the accuracy of hydrological models [45]. The calibration and validation of the SWAT model, made possible by the SWAT Calibration, SWAT-CUP, and the SUFI-2 algorithm, are critical components of this study's methodology. This iterative process of adjusting hydrological parameters to match observed runoff data demonstrates the model's dependability and accuracy. During calibration and validation, performance measures like R², NSE, PBIAS, KGE, and RSR showed sufficient accuracy in modeling runoff. These findings are consistent with Jin (2019) who found similar ranges of model performance metrics in their study of the Yellow River Basin, demonstrating the ability of various rainfall-runoff hydrological models to replicate observed hydrological patterns across diverse geographical settings [46]. During the calibration and validation phases, the identification of essential hydrological parameters (CN, ALPHA_BF, AWC, and EPCO) emphasizes their importance in improving model accuracy. Emphasizing parameters like the curve number (CN) and the baseflow alpha factor (ALPHA_BF) is consistent with the findings of Yasin (2014), who found that these parameters had a significant impact on runoff simulation accuracy in their research of the Tha Chin River Basin [30]. The sensitivity of parameters like as CN, ALPHA_BF, AWC, and EPCO to hydrological processes emphasizes the significance of precise calibration in producing a reliable model simulation. The significance of these elements in impacting the accuracy of hydrological models is consistent with data from other Thai watersheds. For example, Chirachawala (2020) highlighted the sensitivity of hydrological models to these characteristics, in the Yom River Basin with complicated agricultural practices and changing water demands [47]. Their emphasis on curve number and baseflow parameters reinforces the importance of these variables in effectively reproducing runoff and streamflow dynamics in the Mae Khlong River Basin. The sensitivity analysis revealing the importance of SOL_K, SOL_AWC, and GW_DELAY parameters echo the conclusions of the research by Yasin who investigated the hydrological responses of the upper Tha Chin River Basin to study the water quality process showing the effect of groundwater and the soil properties [30]. Their analysis pointed to the significant impact of soil and groundwater parameters on model performance, emphasizing the need for precise calibration in models to reflect the actual hydrological behaviors accurately. The ANN and RF approaches have a propensity to underestimate, in contrast to the SVM approach, which has a more balanced performance. This comparison highlights the potential of ML techniques to improve the accuracy and reliability of hydrological models, a viewpoint shared by Nguyen(2022) emphasizes the effectiveness of ML techniques in improving model predictions in the Mekong Delta [48]. Predicting discharge in ungauged basins is a big difficulty and remains an important topic of hydrology research because of its practical consequences for water resource management, flood risk assessment, and ecological protection. The incorporation of ML techniques—RF, ANN, and SVM—into the SWAT model for discharge prediction in ungauged basins such as Tha Chin represents an innovative strategy for improving model performance and prediction accuracy. This use of ML for regionalization in hydrological modeling, particularly in the setting of ungauged watersheds, provides a novel method for parameter estimate and modification. These contributions are critical in tackling the problems provided by climatic variability and land use changes, hence supporting efforts to improve water resource management. Furthermore, they hold implications for future studies and water management strategies within Thailand and beyond.

## Conclusion

In this study, we employed the SWAT to simulate hydrological processes within the Mae Khlong River Basin, focusing primarily on runoff analysis. The thorough delineation of the river basin into sub-watersheds and HRUs allowed for a detailed investigation of the diverse landscape characteristics, including variations in land use, soil types, and slope gradients. Calibration and validation of the SWAT model using historical data from 1993 to 2017, coupled with the SUFI-2 algorithm, demonstrated the model's capability to accurately reproduce observed runoff patterns. The satisfactory performance metrics obtained during both the calibration and validation phases underscore the reliability and accuracy of the SWAT model in simulating hydrological processes within the Mae Khlong River Basin. Furthermore, the identification and adjustment of key hydrological parameters such as the CN, ALPHA_BF, AWC, and EPCO played a crucial role in enhancing the model's precision. The significance of these parameters in influencing runoff generation and evapotranspiration processes was consistent with findings from previous studies, highlighting their importance in achieving robust and realistic model simulations. Additionally, sensitivity analysis revealed the critical influence of parameters like SOL_K and GW_DELAY on model performance, emphasizing the necessity of careful parameter selection and calibration to improve the accuracy of hydrological models. In addition to simulating hydrological processes within the Mae Khlong River Basin, this study also explored the regionalization of model parameters using ML techniques, namely RF, ANN, and SVM, for discharge prediction in the ungauged Tha Chin River Basin. While all approaches demonstrated utility, the SVM method exhibited superior performance, indicating its potential for accurately predicting hydrological behaviors. These findings highlight the promising role of ML in enhancing the accuracy and reliability of hydrological models, with implications for water resource management strategies not only in Thailand but also in other regions facing similar challenges.

Despite the promising results obtained in this study, several limitations should be acknowledged. Firstly, the accuracy of the simulated hydrological processes is contingent upon the availability and quality of input data, including meteorological, topographic, and land use data. Any inaccuracies or uncertainties in these data can potentially affect the reliability of model simulations. Additionally, the complexity of hydrological processes and the inherent variability in environmental conditions pose challenges in accurately representing these processes within hydrological models. While efforts were made to calibrate and validate the SWAT model, uncertainties still exist, particularly in ungauged or data-scarce regions where validation data is limited. Furthermore, the regionalization of model parameters using ML techniques may introduce additional sources of uncertainty, such as model bias and overfitting, which should be carefully considered in future studies.

This study contributes valuable insights into hydrological modeling techniques and their application in simulating hydrological processes within the Mae Khlong River Basin. The study emphasizes the value of ML for parameter regionalization, crucial for improving hydrological modeling accuracy, especially in ungauged basins. Techniques like Random Forest, Artificial Neural Networks, and Support Vector Machines aid water resource managers in understanding hydrological processes for informed decision-making regarding water allocation and flood forecasting. Sensitivity analyses highlight critical parameters, guiding targeted interventions such as groundwater monitoring or flood mitigation projects. Cross-validation techniques ensure the reliability of regionalized parameters, emphasizing ongoing data refinement efforts to address changing environmental conditions. Future research should focus on advanced ML techniques and interdisciplinary collaborations to foster sustainable water management practices in the Tha Chin River Basin and beyond. Despite the inherent challenges and limitations, the findings highlight the potential of advanced modeling approaches and ML techniques in improving the accuracy and reliability of hydrological models, with implications for water resource management and environmental conservation efforts.

### Limitations

Despite the detailed SWAT modeling and the incorporation of ML techniques for parameter regionalization, this study faces limitations due to potential data inaccuracies, the complexity of hydrological processes, and the challenges of simulating such processes in ungauged basins. Uncertainties from input data quality and ML technique biases may affect the reliability of runoff predictions and require careful consideration. These limitations underscore the importance of continuous model improvement and data enhancement for advancing hydrological simulations and water resource management strategies.

### Future directions

This study highlights the need to improve data accuracy and address hydrological complexity to enhance the reliability of runoff predictions. Future work should focus on expanding data networks, refining the representation of hydrological processes within models, and developing more robust approaches for modeling in ungauged basins.

## Ethics statements

The data used in this research are secondary data derived from the office of the Thai Meteorological Department and Royal Irrigation Department.

## Funding

No Funding was used in this Research.

## CRediT authorship contribution statement

**Phyo Thandar Hlaing:** Conceptualization, Methodology, Software, Formal analysis, Investigation, Writing – original draft. **Usa Wannasingha Humphries:** Formal analysis, Resources, Supervision, Writing – review & editing, Project administration, Funding acquisition. **Muhammad Waqas:** Writing – review & editing.

## Declaration of competing interest

The authors declare that they have no known competing financial interests or personal relationships that could have appeared to influence the work reported in this paper.

## Data Availability

Data will be made available on request.
